# Ultrasound-guided interventions of the upper extremity joints

**DOI:** 10.1007/s00256-022-04148-9

**Published:** 2022-08-13

**Authors:** Rina P. Patel, Kevin McGill, Daria Motamedi, Tara Morgan

**Affiliations:** Department of Radiology and Biomedical Imaging, University of California, San Francisco, 500 Parnassus Avenue, San Francisco, CA 94143 USA

**Keywords:** Glenohumeral joint, Acromioclavicular joint, Sternoclavicular joint, Elbow joint, Radiocarpal joint, Trapeziometacarpal joint, Ultrasound, Corticosteroid injection, Arthrography, Aspiration

## Abstract

**Abstract:**

Ultrasound guidance is valuable for performing precise joint interventions. Joint interventions may be requested for therapeutic and diagnostic pain injections, joint aspiration in the setting of suspected infection, or contrast injection for arthrography. In practice, interventions of the shoulder girdle, elbow, and hand/wrist joints may be performed without any imaging guidance. However, imaging guidance results in more accurate interventions and better patient outcomes than those performed by palpation alone. When compared to other modalities used for imaging guidance, ultrasound has many potential advantages. Radiologists should be prepared to perform ultrasound-guided upper extremity joint interventions utilizing recommended techniques to optimize clinical practice and patient outcomes.

**Key points:**

1. Ultrasound-guided injections of the glenohumeral, acromioclavicular, sternoclavicular, elbow, and hand/wrist joints have higher accuracy than injections performed without imaging guidance.

2. Ultrasound-guided aspirations of upper extremity joints have advantages to fluoroscopic-guided aspirations because of the potential to identify effusions, soft tissue abscess, or bursitis.

3. Ultrasound-guided contrast injection prior to MR arthrography is as accurate as fluoroscopic-guided injection for upper extremity joints.

## Introduction

Upper extremity joint procedures are commonly requested and include corticosteroid injection for pain, contrast injection for arthrogram, or joint aspiration. Upper extremity joint interventions have been described with or without imaging guidance utilizing a variety of imaging modalities, including fluoroscopy, computed tomography (CT), magnetic resonance imaging (MRI), and ultrasound. Interventions of upper extremity joints are often performed without imaging guidance by using palpation of anatomic landmarks [1]. However, numerous studies show higher accuracy of image-guided over palpation-guided injections, including for the small joints of the upper extremity [2–9].

Ultrasound-guided joint procedures have many advantages in addition to higher accuracy [10, 11]. Sonographic assessment of the joint can be rapidly performed prior to the procedure and can identify soft tissue abnormalities that could affect procedural technique, such as fluid collections, synovitis, or anatomic variations. These findings are likely to be missed using fluoroscopy or without imaging guidance. This is particularly valuable when there are obstructing deformities at the joint space, such as large osteophytes, or when there is clinical concern for soft tissue infection which may change the procedural approach. Identification of alternate sources of infection outside of the joint space, such as abscess or bursitis, allows for a rapid pivot from aspiration of the joint to aspiration of other adjacent fluid collections. Ultrasound also lacks the ionizing radiation associated with fluoroscopic or CT-guided procedures. Finally, easy manipulation of transducer placement can allow for more flexible and comfortable positioning for the patient.

The purpose of this review is to discuss common procedural techniques to access each joint for therapeutic injection, contrast injection, or joint aspiration. The accuracy of ultrasound-guided procedures compared to alternative methods will be reviewed. Some of the techniques will be universal for all joints described. For most upper extremity joint injections, a 9-MHz or 15-MHz linear transducer may be used. A smaller footprint linear array 18-MHz transducer, or “hockey stick” transducer, may be used for the acromioclavicular, sternoclavicular, or elbow joints and is routinely used for the small joints of the hand/wrist. Prior to all needle interventions, local anesthetic is used to anesthetize the skin and needle track. The discussion of therapeutic injections will focus on the most common medications requested—corticosteroids and anesthetic. The type and volume of corticosteroid and anesthetic injected are based on operator or ordering physician preference. Other materials, such as hyaluronic acid and platelet-rich plasma, have also been injected into joints for pain, though the data on efficacy of hyaluronic acid injection in upper extremity joints is limited and outside the scope of this review [12].

Joint aspiration may be requested to diagnose septic arthritis or crystalline arthropathy. In most cases, the needle approach for injections and aspirations is similar, but where relevant, the differences in technique will be highlighted. With joint aspiration, potential sources of overlying soft tissue infection, such as bursitis or intramuscular abscess, should be avoided to prevent potential seeding of the joint with infected tissue. A larger needle (16- to 18-gauge) is often necessary for successful aspiration of viscous joint fluid, compared to smaller 22- or 25-gauge needles used for therapeutic injection. When aspiration is unsuccessful (“dry tap”), saline lavage may be considered. Saline lavage can improve yield for diagnosing periprosthetic joint infections or septic arthritis at the hip [13, 14]. In some cases, image-guided synovial biopsy may be performed to diagnose joint infection. Synovial biopsy has been shown to have similar diagnostic accuracy as synovial aspiration for diagnosis of periprosthetic joint infections at the hip and knee [15] and may be considered when aspiration is not successful. The decision to perform joint lavage and/or synovial biopsy often varies with institution.

Ultimately, whether ultrasound-guided upper extremity joint interventions are requested will depend on access to equipment for imaging guidance, experience and preference of the ordering provider, and indication for the procedure. Radiologists should be proficient and prepared to perform these interventions when requested.

## Glenohumeral joint

### Indications

Percutaneous access of the glenohumeral joint is commonly performed for therapeutic and diagnostic injection, as well as for aspiration. Systematic reviews of glenohumeral joint injections/aspirations show that accuracy is significantly higher for image-guided compared to non-image-guided techniques [2, 4, 8]. Although the average accuracy for non-image-guided techniques is moderately high, ranging from 72.5–79%, the reported range of accuracy is very variable, ranging from 27–100% [2, 4, 8]. On the contrary, image-guided glenohumeral joint injection has proven to be both more accurate and precise with an average accuracy of 92.5–95% and both a narrower and higher range of accuracy of 83–100% [2, 4, 8].

Corticosteroid injections at the shoulder are widely performed to treat shoulder pain. One of the most common indications for intra-articular injection into the glenohumeral joint is adhesive capsulitis or frozen shoulder [16]. Adhesive capsulitis is an idiopathic condition characterized by pain and restricted range of motion without radiographic abnormality or identifiable underlying cause [16]. Non-operative treatment of adhesive capsulitis is most common and includes nonsteroidal anti-inflammatory medications, physical therapy, intra-articular corticosteroid injection with or without hydro-dilation [16]. The benefit of hydro-dilatation for adhesive capsulitis is debated. Corticosteroid injection has positive outcomes, although no significant benefit of high versus low volume injectate has been shown [17, 18]. If hydro-dilatation is performed, then anterior injection may have better outcomes than via the posterior approach. A comparison of posterior versus anterior injection with hydro-dilatation for adhesive capsulitis showed significantly greater improvement in range of motion and decrease in pain for those with anterior injections [19].

Ultrasound guidance is also indicated for contrast injection into the glenohumeral joint prior to magnetic resonance arthrography (MRA). Direct contrast injection can be performed under fluoroscopic guidance and ultrasound guidance or by palpation. Both fluoroscopic and ultrasound guidance have high accuracy (up to 100%) and are preferred over non-image-guided injection [20]. The accuracy of fluoroscopic versus ultrasound-guided contrast injection is comparable, although ultrasound has the advantage of lack of radiation and may be better tolerated. Two studies comparing ultrasound versus fluoroscopic-guided contrast injection showed significantly shorter procedure times and lower reported pain for ultrasound-guided procedures [20, 21].

Ultrasound guidance may be requested for glenohumeral joint aspiration and has potentially significant advantages over fluoroscopic guidance or non-image guidance. Joint effusions can be directly seen and targeted with ultrasound guidance and other concomitant or alternate sources of infection, such as abscess or bursitis can also be identified. Although septic arthritis of the shoulder is not as common as hip or knee septic arthritis [22], joint aspiration remains a key part of decision-making on surgical intervention [22, 23].

### Procedure technique

Prior to ultrasound-guided intervention, sonographic evaluation of the shoulder should be performed. Preprocedural imaging of the soft tissues is a major advantage of ultrasound compared to fluoroscopy. Findings on preprocedural imaging can aid or alter the best patient positioning and approach.

**Fig. 1:**
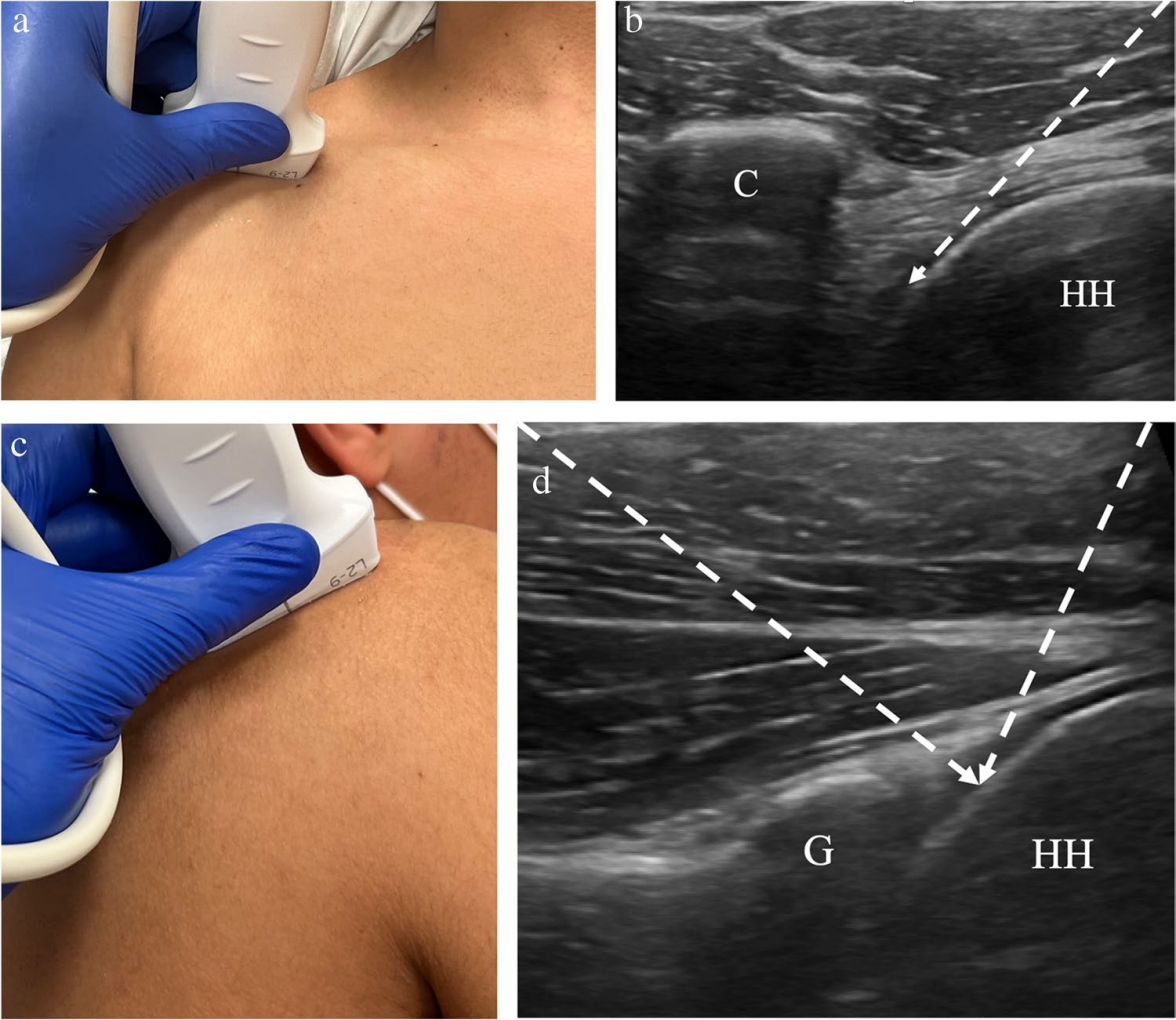
Technique for glenohumeral joint injection. **a** Image of a healthy volunteer shows transducer placement for anterior approach, which yields ultrasound image of the anterior glenohumeral joint space (**b**), showing the coracoid (C) and humeral head (HH) with expected needle trajectory (dashed arrow). **c** Image of a healthy volunteer shows transducer placement for posterior approach, which yields ultrasound image of the posterior glenohumeral joint space (**d**), showing the glenoid (G) and humeral head (HH) with the expected needle trajectory for either the medial-to-lateral or lateral-to-medial approach (dashed arrows)

**Fig. 2:**
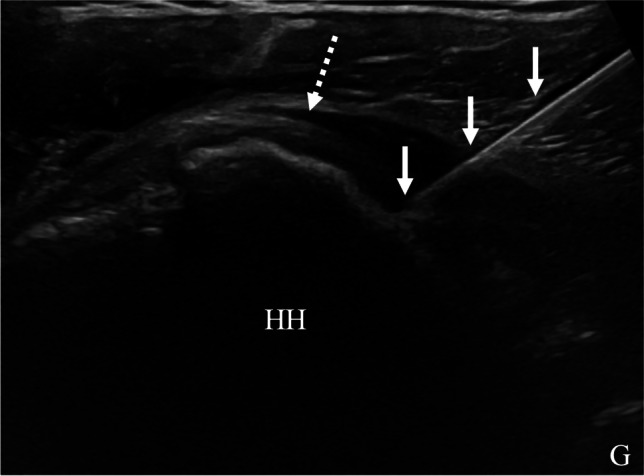
Ultrasound image of glenohumeral joint corticosteroid injection using posterior approach in a 46-year-old female with chronic shoulder pain and history of partial tear of the supraspinatus tendon. The humeral head (HH) and glenoid (G) are visualized posteriorly. The needle (arrows) trajectory extends medial to lateral with the tip at the humeral head, deep to the infraspinatus tendon (dashed arrow)

The glenohumeral joint may be accessed via an anterior or posterior approach. When using the posterior approach, the patient is in a lateral decubitus position contralateral to the affected shoulder (Fig. 1). Preprocedural imaging should be performed to identify the site for skin puncture and approach. The transducer is positioned over the posterior aspect of the joint, at the midpoint of the humeral head where the glenoid rim should be well-visualized. A 22-gauge or 25-gauge needle is typically used to access the glenohumeral joint for injection. The needle is inserted into the glenohumeral joint towards the humeral head either medial to lateral or lateral to medial. The needle should pass through the infraspinatus tendon and joint capsule, with the needle tip located on the humeral articular cartilage. The needle tip should be sufficiently visualized within the joint to avoid inadvertent extra-articular injection (Fig. 2). When using the anterior approach, the patient is positioned supine with the arm in slight external rotation [24]. The transducer is positioned to visualize the coracoid medially and the superomedial humeral head laterally (Fig. 1). The needle is advanced lateral to medial and should pass behind the subscapularis tendon to reach the medial humeral cartilage [21, 24].

Intra-articular position of the needle should be confirmed visually prior to injection. Initial injection of anesthetic can demonstrate flow of injectate into the joint and further confirm intra-articular position. Once intra-articular position is confirmed, the corticosteroid and any additional anesthetic can be injected. As discussed above, a higher volume injectate, with or without saline, may be used in patients with adhesive capsulitis, although without clear benefit. Typical volumes for glenohumeral joint injection range from 8–12 mL volume. Studies evaluating the use of hydro-dilatation for adhesive capsulitis have reported injection volumes of up to 17–18 mL [18, 19]. The recommended volumes for injection into the other upper extremity joints are listed in Table 1. While injecting, continuous sonographic visualization should demonstrate flow of the injectate into the joint space. The injectate flows into the dependent portion of the joint, so capsular distention may not be visualized.

**Table 1:** Suggested volumes to be injected into the upper extremity joints

Joint	Total volume
Glenohumeral	8–12 mL
Acromioclavicular	1–2 mL
Sternoclavicular	0.5–2 mL
Elbow	3–8 mL
Wrist (radiocarpal)	2–5 mL
Small joints of hand, including thumb carpometacarpal	0.25–1 mL

Lower volumes may be used for therapeutic injections. Higher volumes may be needed for joint distention prior to MR arthrography. Higher volumes than those described above may be used, though there is potential risk for extravasation of injected fluid

The technique for contrast injection prior to MRA is very similar to that for corticosteroid injection. Ultrasound-guided posterior and anterior approaches are both successful for contrast injection and which is more beneficial is debated. In one comparison of ultrasound-guided anterior and posterior approaches for arthrography, the anterior approach was more successful with the first attempt and had shorter mean procedural time [21]. In another study, there was no significant difference in procedural time comparing the anterior and posterior approach, although fewer incidences of contrast extravasation occurred with the posterior approach [25]. Given the potential for contrast extravasation, a tailored approach depending on the indication for arthrography may be the best method.

**Fig. 3:**
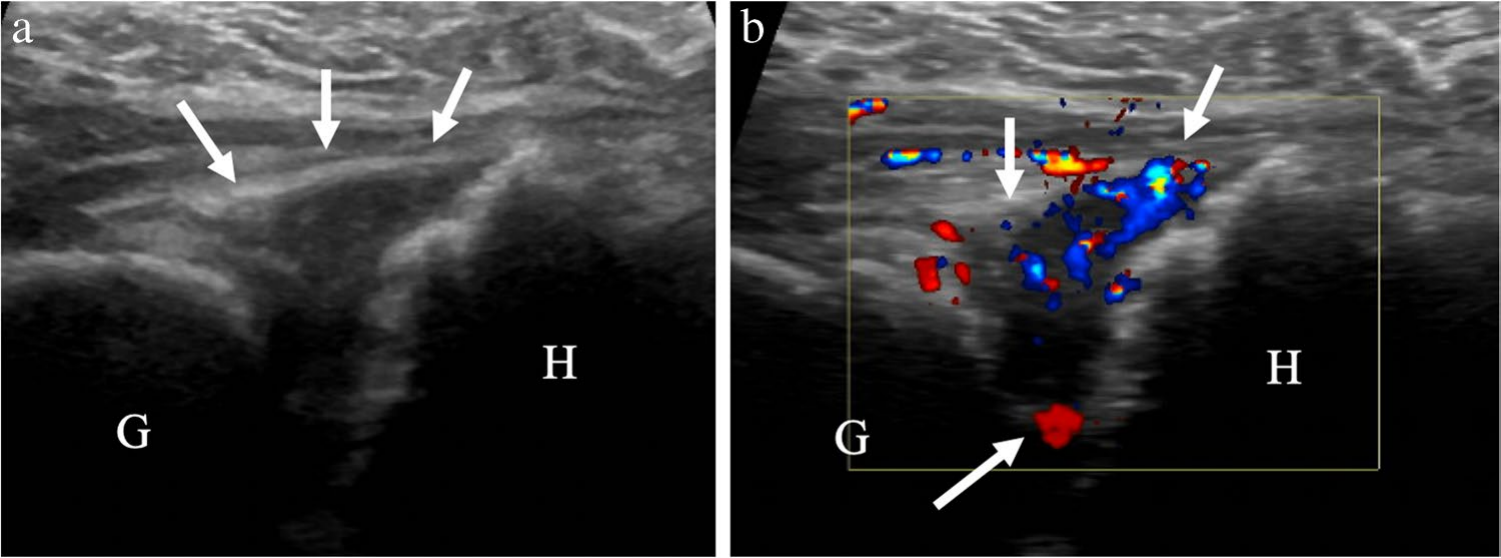
73-year-old female with neck pain and elevated erythrocyte sedimentation rate (ESR) and c-reactive protein (CRP), as well as total shoulder arthroplasty placement one month prior. Point of care ultrasound in the emergency department described large glenohumeral joint effusion, so aspiration was requested. **a** Ultrasound image shows hypoechoic capsular distention (arrows) between the humeral (H) and glenoid (G) components. **b** Color Doppler image shows hypervascularity within the joint (arrows), consistent with synovitis. No fluid was detected, so aspiration was not performed. Subsequent cervical spine imaging identified discitis-osteomyelitis of the cervical spine as the source of infection

The approach for ultrasound-guided glenohumeral joint aspiration is modified to target the site of maximal joint distention. Compared to fluoroscopic or palpation-guided intervention, ultrasound guidance can identify whether effusion is present, thereby avoiding unnecessary aspiration attempts (Fig. 3). Although lavage has been shown to be efficacious for diagnosing periprosthetic joint infection, whether lavage increases diagnostic yield in the glenohumeral joint is debatable and at least one study shows no significant increase in yield following irrigation of 10 mL of saline into the glenohumeral joint [23]. The decision to perform joint irrigation for suspected infection will depend on institutional preference.

## Acromioclavicular joint

### Indications

Acromioclavicular (AC) joint injection and aspiration are less commonly indicated than glenohumeral joint interventions, and research on ultrasound-guided acromioclavicular joint injections is limited. The AC joint is often a source of shoulder pain, either in isolation or in combination with rotator cuff tendon pathology. Treatment of AC joint pain depends on whether there is isolated AC joint osteoarthritis or associated rotator cuff tendon tear and/or subacromial/subdeltoid bursitis. Corticosteroid injection into the AC joint can be effective for short-term pain relief in those with AC joint osteoarthritis with or without rotator cuff pathology and for long-term pain relief in those with isolated AC joint osteoarthritis [26].

Access of the AC joint using palpation and anatomic landmarks alone has variable accuracy and can be as low as 36.5% [7, 27]. Image-guided AC joint injections are significantly more accurate, with a reported average accuracy of 100% for fluoroscopic-guided injections [4]. Ultrasound guidance significantly improves accuracy to 96% [26]. Another comparison of AC corticosteroid injection by ultrasound guidance versus palpation showed significantly greater improvement in functional status and decrease in pain when ultrasound guidance was used [6]. There has been no direct comparison of ultrasound versus fluoroscopic-guided AC joint interventions, although there are aforementioned benefits of using ultrasound guidance for joint aspiration.

Septic arthritis of the acromioclavicular joint is less common than septic arthritis of the glenohumeral joint. Risk factors for AC joint septic arthritis include diabetes mellitus, intravenous drug use, immunocompromised state, or local trauma [28]. Although there is no standard treatment algorithm for AC joint septic arthritis, aspiration can be helpful in confirming infection and culture of specific organisms [28]. Clinically, septic arthritis of the AC joint may be difficult to distinguish from infectious subacromial/subdeltoid bursitis or other soft tissue infection. In these cases, preprocedural ultrasound imaging may be valuable in identifying presence or absence of effusion, subacromial/subdeltoid bursitis, erosion, or concomitant glenohumeral joint effusion [29].

### Procedure technique

**Fig. 4:**
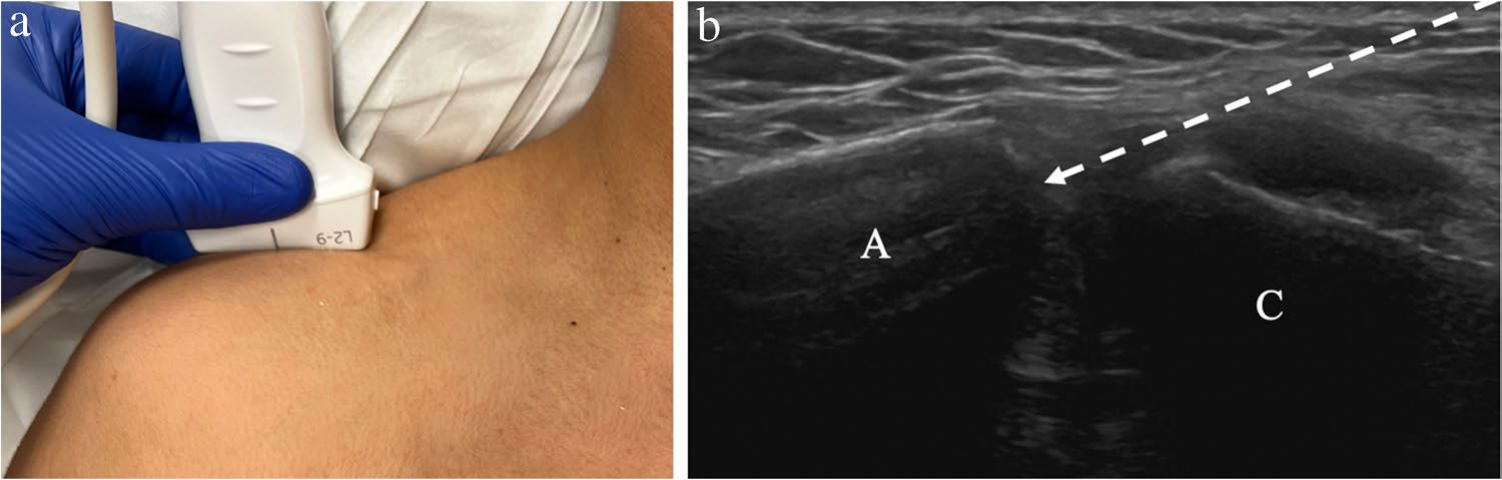
Technique for acromioclavicular joint injection. **a** Image of a healthy volunteer shows transducer placement, which yields ultrasound image of the acromioclavicular joint space (**b**), showing the acromion (A) and clavicle (C) with expected needle trajectory for in-plane approach (dashed arrow)

**Fig. 5:**
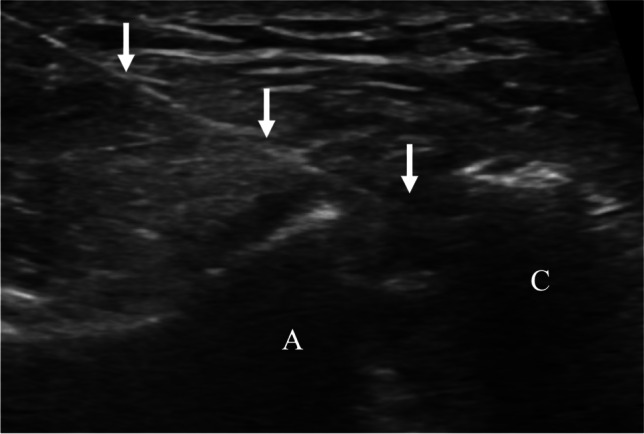
77-year-old female with chronic shoulder pain and tenderness to palpation over the acromioclavicular joint, for which corticosteroid injection was requested. Ultrasound image of acromioclavicular corticosteroid injection with the in-plane approach shows needle trajectory (arrows) with tip in the joint space between the acromion (A) and clavicle (C)

For ultrasound-guided AC joint injections, the patient is in the seated, upright position with the arm resting by the side. Preprocedural imaging should be performed to identify abnormalities of the AC joint and plan approach. The AC joint may be accessed via an in-plane or out-of-plane technique. Using either technique, the transducer is placed parallel with the clavicle over the superior margin of the joint space (Fig. 4). The curved surfaces of the acromion and clavicle should be visualized, as well as the anechoic joint space between. With the in-plane technique, the needle is inserted at either the medial or lateral margin of the transducer and advanced obliquely into the joint (Fig. 5). With this technique, the entire length of the needle should be visualized. With the out-of-plane technique, the needle is inserted at the midpoint of the transducer and advanced vertically into the joint space. With this technique, only the short axis of the needle is visualized as an echogenic dot. The in-plane approach can be challenging when bony prominences or osteophytes are present, however, careful planning with preprocedural screening can identify a clear path for the needle. Intra-articular position is confirmed when the needle tip is visualized within the anechoic joint space between the acromion and clavicle. If the indication is for therapeutic injection, then the operator should be cautious with the volume injected. The AC joint typically holds approximately 1–2 mL of fluid (Table 1) [26]. Larger volume injection could cause discomfort and may result in extravasation of medication into the surrounding soft tissues.

## Sternoclavicular joint

### Indications

The sternoclavicular (SC) joint is formed by two compartments separated by an articular disk. The indications for SC joint intervention are like that of the other small joints, though non-traumatic pathology of the SC joint is comparatively uncommon. Pain at the SC joint may be related to a variety of pathology, including osteoarthritis, inflammatory arthropathy, or crystalline arthritis. In patients with SC joint pain related to osteoarthritis, intraarticular corticosteroid injection can be valuable for pain reduction [30].

Sternoclavicular joint intervention by palpation and anatomic landmarks can be accurate. One study of injections in cadavers showed an overall accuracy of 78% [31], although accuracy of ultrasound-guided injections is up to 100% [32]. Studies directly comparing different methods of intervention at the SC joint are limited. Image-guided SC joint interventions have been described by CT [33], fluoroscopic or ultrasound guidance.

Aspiration of the SC joint for suspected septic arthritis may be requested to confirm the diagnosis and guide treatment planning. Although SC septic arthritis is uncommon, it has been associated with intravenous drug use, as well as non-drug-use-related risk factors such as diabetes mellitus [34]. Although there are no studies examining the utility of ultrasound-guided aspiration for diagnosis of SC joint septic arthritis, there is one study on utility of CT-guided aspiration [35]. In this study, Taneja et al. [35] found that CT-guided SC joint intervention yielded positive cultures in 52% of patients, though this was based on aspiration, biopsy, or a combination of both techniques.

### Procedure technique

**Fig. 6:**
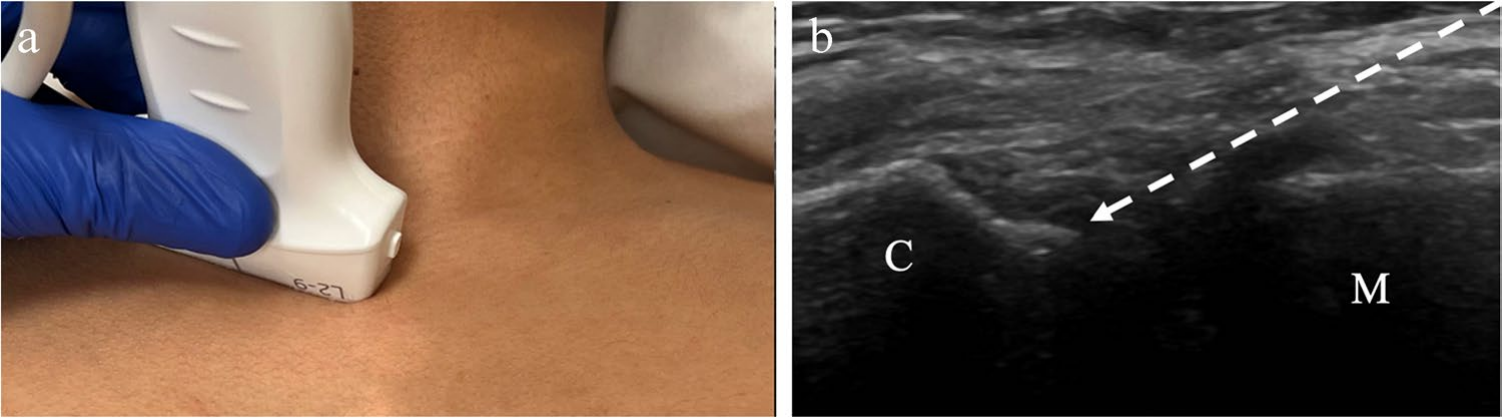
Technique for sternoclavicular joint injection. **a** Image of a healthy volunteer shows transducer placement, which yields ultrasound image of the sternoclavicular joint space (**b**), showing the clavicle (C) and manubrium (M) with expected needle trajectory for in-plane approach (dashed arrow)

**Fig. 7:**
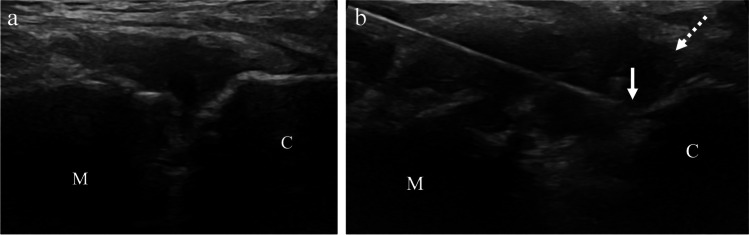
66-year-old male with right sternoclavicular joint pain that worsens after playing racquetball, for which corticosteroid injection was requested. **a** Preprocedure ultrasound image shows the sternoclavicular joint with the manubrium (M) and clavicle (C). **b** Intra-procedure ultrasound image shows needle tip (arrow) at the clavicle (C) and fluid distention of the joint near the clavicle (dashed arrow)

For ultrasound-guided sternoclavicular joint injections, the patient is in the supine or semi-upright position with the arms resting by the sides. The transducer is placed along the long axis of the medial clavicle and then moved medially until the sternoclavicular joint is visualized (Fig. 6). The clavicular margin is visualized laterally, and the manubrium medially and inferior to the clavicle. Following preprocedural imaging assessment for joint abnormalities, including effusion, the approach should be determined. The needle is inserted in an in-plane approach from medial to lateral, to avoid the bony protuberance of the slightly superficial clavicle (Fig. 7). The intra-articular disk that separates the clavicular and sternal compartments may or may not be identified. In older patients, this disk may be degenerated, allowing for flow of injectate from one compartment to the other. In younger patients, the disk may be intact and block flow of injectate into both compartments [32]. The utility of targeting each joint compartment has not been studied. The volume of fluid that can be injected into the SC joint is small (Table 1). Studies of single target injections into the SC joint show that the joint can accommodate a range of 0.5–2.0 mL for injection [32, 33].

## Elbow joint

### Indications

Ultrasound-guided procedures around the elbow are commonly performed, though often the indications are for treatment of ligament or tendon abnormalities rather than intra-articular procedures. Primary osteoarthritis of the elbow is uncommon compared to osteoarthritis at other joints, so intra-articular injection for elbow osteoarthritis is infrequently requested. Corticosteroid injections are the most common type of intra-articular injection described at the elbow, although hyaluronic acid preparations may also be administered for treatment of osteoarthritis. The elbow may also be affected by inflammatory arthritis or crystalline arthropathy, whether this is at the joint or at the olecranon bursa. Intra-articular corticosteroid injections for the pain related to inflammatory arthritis may be performed but are typically secondary to or in addition to treatment with disease-modifying antirheumatic drugs [36].

Intra-articular injections of the elbow may be performed by palpation of anatomic landmarks, although the reported range of accuracy is wide and potentially lower than when ultrasound guidance is used. The accuracy for ultrasound-guided injection ranges from 91–100 versus 64–100% [1, 5, 37] without imaging guidance. Ultrasound-guided aspiration of the elbow may be requested when septic arthritis or crystalline arthropathy is suspected. Ultrasound is well-suited to detect elbow joint effusion [38], and at least one study that included elbow and small joints found that ultrasound-guided aspirations were more successful than non-image guided aspiration (97% success versus 32% for palpation-guided) [3]. Ultrasound guidance may also be used for contrast injection into the elbow prior to MR arthrogram, although there are limited studies of the accuracy of ultrasound versus fluoroscopic guidance for this indication.

### Procedure technique

**Fig. 8:**
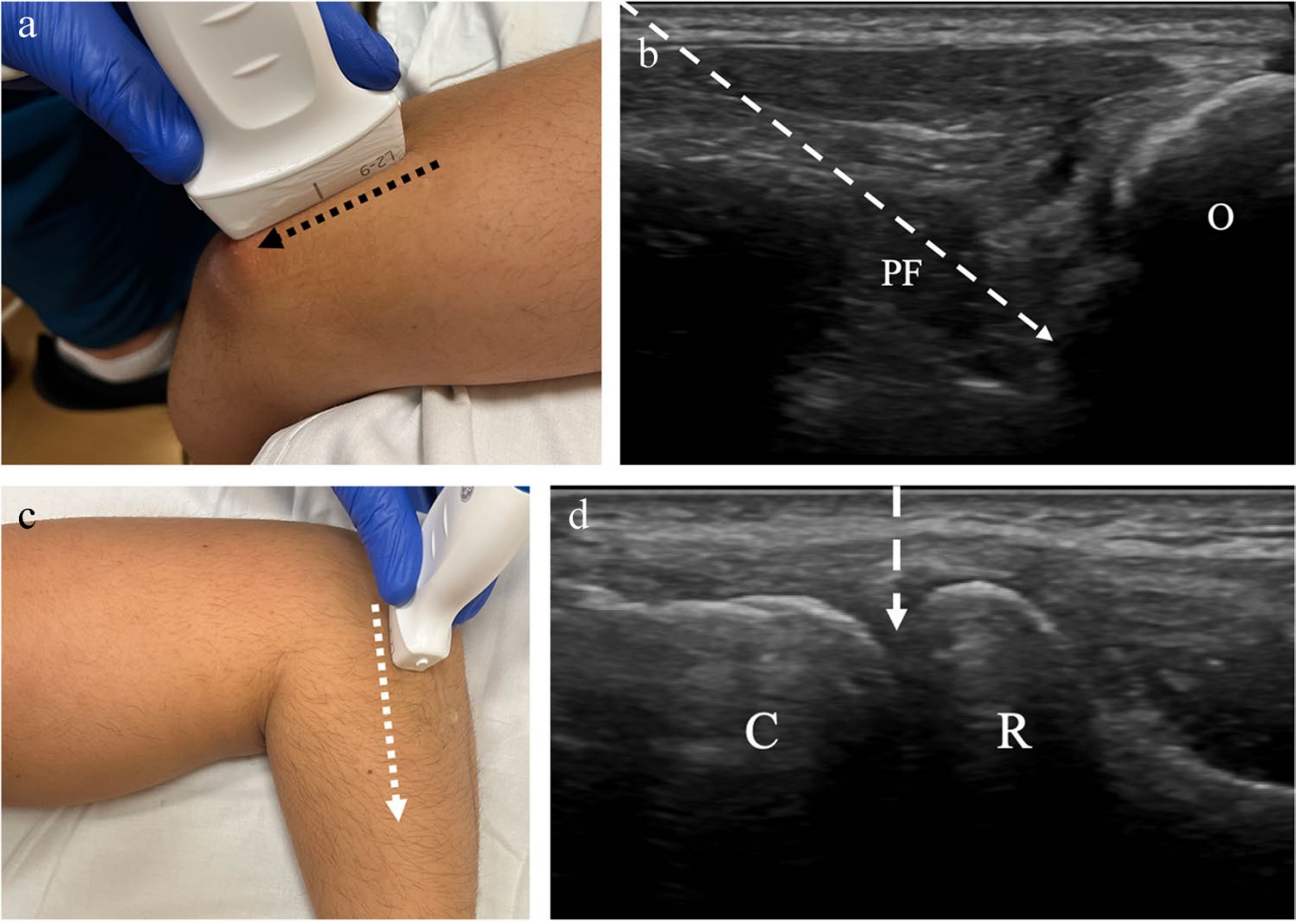
Technique for elbow joint injection. **a** Image of a healthy volunteer shows transducer placement for posterior approach. The transducer is oriented along the triceps tendon insertion at the olecranon (black dashed arrow) and translated laterally, which yields ultrasound image of the olecranon (O) and posterior fat pad (PF) (**b**). The needle trajectory (dashed arrow) is shown from proximal to distal. **c** Image of a healthy volunteer shows transducer placement for lateral, radiocapitellar approach. The transducer is oriented along the long axis of the radius (dashed arrow) which yields ultrasound image of the radiocapitellar joint space (**d**), showing the radial head (R) and capitellum (C) with expected needle trajectory for out-plane approach (dashed arrow)

**Fig. 9:**
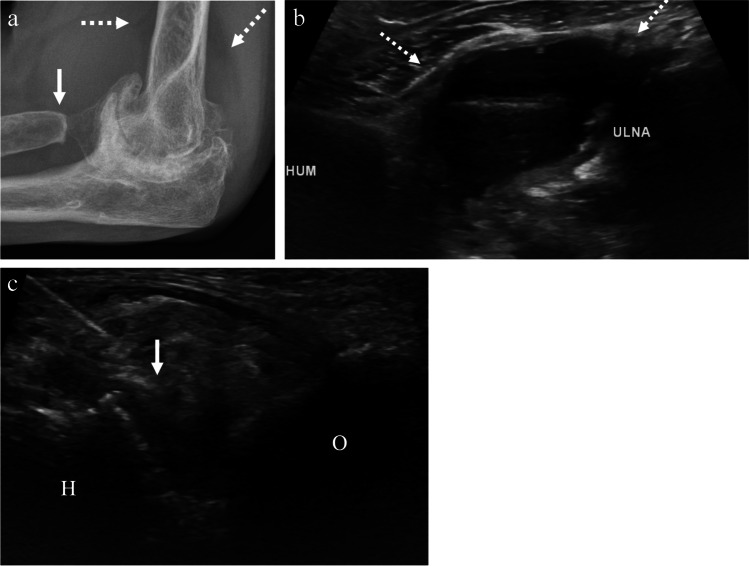
A 64-year-old female with a history of old elbow trauma followed by multiple elbow surgeries, including radial head resection, now with severe secondary osteoarthritis. **a** Lateral radiograph of the elbow shows severe joint space narrowing and osteophytosis, as well as radial head resection (arrow) and large effusion with elevation of the elbow fat pads (dashed arrows). After excluding infection of the joint, ultrasound-guided corticosteroid injection was requested for therapeutic pain relief. **b** Preprocedure ultrasound image at the posterior joint space shows large effusion with synovitis (dashed arrows). A posterior, transtriceps approach was used to aspirate joint fluid and inject corticosteroid and anesthetic. **c** Intraprocedure ultrasound shows the humerus (H) and olecranon (O) posteriorly with the needle tip (arrow) within the area of synovitis at the posterior joint space

Multiple methods for accessing the elbow joint have been described [39–41]. With the posterior, trans-triceps approach, the patient is in the prone position with the elbow flexed at 90° and the forearm hanging off the examination table (Fig. 8). The transducer is placed in a long axis over the distal triceps tendon insertion at the olecranon fossa. The transducer is then moved laterally just past the lateral margin of the tendon. The olecranon, posterior fat pad, and humerus should be visualized. The needle is inserted at the lateral margin of the tendon and passed in-plane with the transducer until the needle tip reaches the posterior margin of the humerus, deep to the posterior fat pad (Fig. 9). The medial margin of the triceps tendon should be avoided to avoid inadvertent contact of the ulnar nerve in the cubital tunnel. With the lateral approach, the patient is seated next to the table or prone with the arm resting on the examination table. The elbow is flexed at 90° and the wrist/forearm is rotated so that the thumb is pointing up. The transducer is placed at the elbow along the longitudinal axis of the radius (Fig. 8). In this position, the radiocapitellar joint space should be visualized. The needle may be inserted into the radiocapitellar joint space by an in-plane or out-of-plane approach. Either lateral or posterior approach may be used prior to contrast injection for MR arthrogram of the elbow. The volume of contrast injection into the elbow that has been described ranges from 2 to 14 mL [42–44]. Although, injection greater than 8 mL may be associated with contrast extravasation [39]. For joint aspiration, the approach that allows for optimal visualization of joint effusion should be used.

## Wrist/hand joints

### Indications

Intra-articular corticosteroid injections are frequently performed in the hand for osteoarthritis [37, 45, 46], though the benefit of corticosteroid injection compared to other treatment options is unclear [47, 48]. Similarly, corticosteroid injections for the pain related to inflammatory arthritis may be performed but are typically secondary to or in addition to treatment with disease-modifying antirheumatic drugs [36, 49]. Hyaluronic acid injections may be beneficial in patients with thumb carpometacarpal or trapeziometacarpal joint osteoarthritis [45, 50], and may provide additional benefit when combined with corticosteroid injection [51]. These injections are often performed without image guidance using palpable landmarks. However, in one cadaver study [9], thumb carpometacarpal joint injections were significantly more accurate with ultrasound guidance (38% without vs. 72% with ultrasound). Ultrasound guidance is also used for contrast injection prior to MR arthrogram of the wrist. One study of contrast injections found that ultrasound-guided injections were significantly more accurate than those done by palpation (100% vs 72%) [52]. Although ultrasound and fluoroscopy had similar accuracy of 100%, ultrasound-guided injections were significantly shorter and less painful than fluoroscopic-guided injections [52]. Ultrasound guidance may also be advantageous in patients with significant deformity or narrowing of the joint space from underlying arthritis.

### Procedure technique

**Fig. 10:**
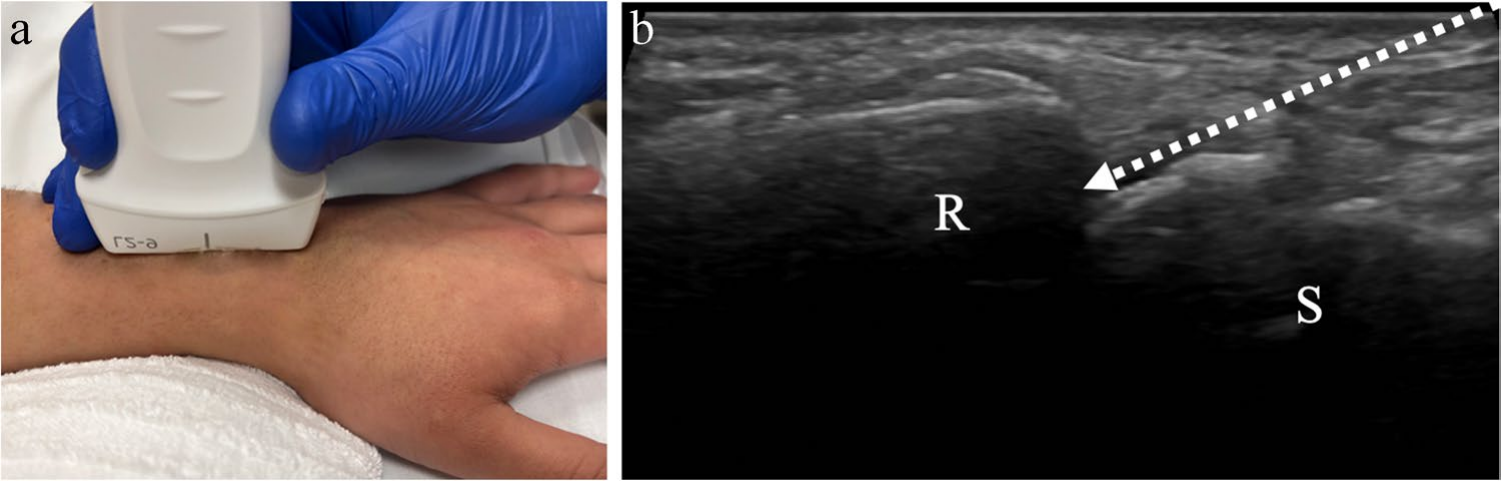
Technique for wrist or radiocarpal joint injection. **a** Image of a healthy volunteer shows transducer placement for radiocarpal joint approach. The transducer is oriented along the long axis of the distal radius which yields ultrasound image of the radiocarpal joint space (**b**), showing the distal radial epiphysis (R) and scaphoid (S) with expected needle trajectory for in-plane approach (dashed arrow)

**Fig. 11:**
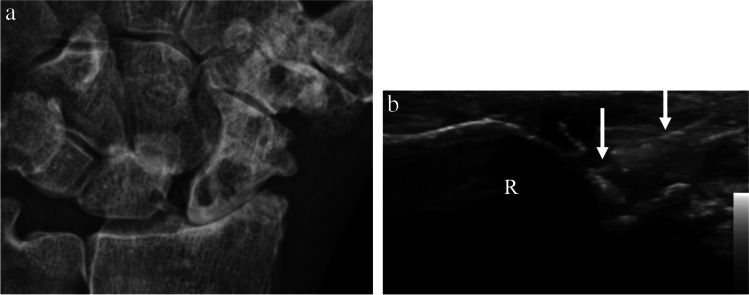
A 84-year-old male with chronic wrist pain for ten years. **a** Posteroanterior radiograph of the left wrist shows severe radiocarpal joint space narrowing with osteophytosis and cystic change in the radius and scaphoid. Ultrasound-guided corticosteroid injection was requested for therapeutic pain relief. **b** Intraprocedure ultrasound image shows the distal radius (R) at the radiocarpal joint with needle (arrows) in the dorsal joint space

The hand and wrist are comprised of multiple joints with varying anatomy. At the wrist, the radiocarpal, distal radioulnar, or midcarpal joint compartments may be injected. Technique will vary depending on the joint in question; therefore, the discussion below will focus on two of the most commonly requested by ultrasound guidance: radiocarpal and thumb carpometacarpal. For ultrasound-guided radiocarpal joint interventions, the hand is placed palm down on the table and the transducer is placed along the longitudinal axis of the distal radius, centered over the radiocarpal joint space (Fig. 10). The distal radial epiphysis and first carpal row should be visualized. The needle may be inserted in-plane from distal to proximal into the radiocarpal joint (Fig. 11). Alternatively, the needle may be inserted out-of-plane to the transducer vertically into the joint space. As described above, with the out-of-plane technique, only the short axis of the needle tip is visualized as it enters the joint space. This technique is challenging, as the short axis of the needle tip may be difficult to distinguish from the remainder of the needle length. However, the in-plane technique has some disadvantages in the hand/wrist where there is limited soft tissue around the joints through which the needle can traverse and where a long needle course can be painful. For contrast injection prior to arthrography, a suggested volume of approximately 2–5 mL of contrast is injected (Table 1) [42, 43, 53].

**Fig. 12:**
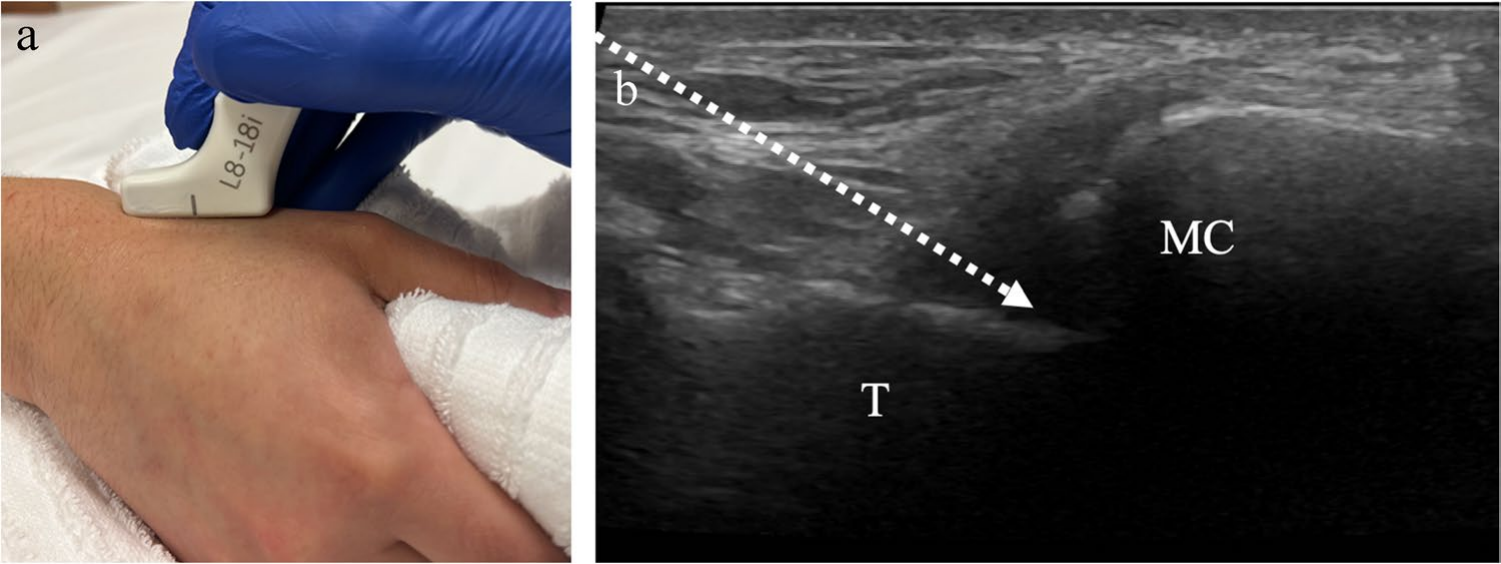
Technique for thumb carpometacarpal joint injection. **a** Image of a healthy volunteer shows the hand in a fist position with the ulna against the table. The transducer is oriented along the long axis of the thumb metacarpal base which yields ultrasound image of the joint space (**b**), showing the trapezium (T) and thumb metacarpal (MC) with expected needle trajectory for in-plane approach (dashed arrow)

**Fig. 13:**
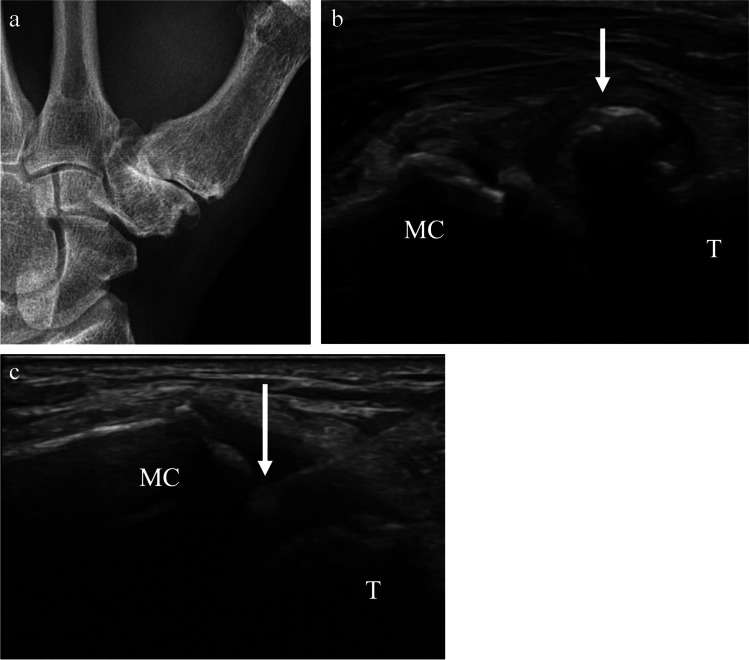
A 67-year-old male with severe thumb carpometacarpal joint osteoarthritis. **a** Posteroanterior radiograph of the left wrist shows severe thumb carpometacarpal joint space narrowing with large osteophytes. The patient had had prior injections by palpation, which were not effective for pain control. Ultrasound-guided corticosteroid injection was requested for pain relief. **b** Preprocedure ultrasound image shows obstructing ossification (arrow) at the joint. **c** Following pre-procedural assessment, an accessible path was found between the trapezium (T) and thumb metacarpal (MC), and intraprocedure ultrasound images shows the needle tip (arrow) in the joint space

For ultrasound-guided thumb carpometacarpal (or trapeziometacarpal) joint injections, the hand is positioned in a fist with the ulna against the table and radial side facing up towards the operator. The transducer is placed along the long axis of the thumb and the trapezium and proximal metacarpal should be visualized (Fig. 12). The needle may be inserted out-of-plane vertically into the joint or via the in-plane approach from proximal to distal (Fig. 13). The volume that can be injected into the small joints of the hand has not been well studied, though reported volumes range from 0.25 to 1 mL (Table 1) [54]. For ultrasound-guided aspirations, the approach that best visualizes joint fluid should be employed.

## Conclusion

Imaging guidance with ultrasound is valuable for interventions of the shoulder girdle, elbow, and hand/wrist. Although small joint injections may be performed without imaging guidance, ultrasound guidance provides for significantly higher accuracy at multiple joints of the upper extremity. Furthermore, ultrasound has many potential advantages over other forms of imaging guidance, especially in the setting of possible joint infection. Fully understanding and appreciating the many utilities of ultrasound for image-guided interventions of the upper extremity is crucial for the practicing radiologist.
